# Numerical stress analysis of the iris tissue induced by pupil expansion: Comparison of commercial devices

**DOI:** 10.1371/journal.pone.0194141

**Published:** 2018-03-14

**Authors:** Royston K. Y. Tan, Xiaofei Wang, Shamira A. Perera, Michaël J. A. Girard

**Affiliations:** 1 Ophthalmic Engineering & Innovation Laboratory, Department of Biomedical Engineering, Faculty of Engineering, National University of Singapore, Singapore, Singapore; 2 Singapore Eye Research Institute, Singapore National Eye Centre, Singapore, Singapore; 3 Duke-NUS, Singapore, Singapore; Politecnico di Milano, ITALY

## Abstract

**Purpose:**

(1) To use finite element (FE) modelling to estimate local iris stresses (i.e. internal forces) as a result of mechanical pupil expansion; and to (2) compare such stresses as generated from several commercially available expanders (Iris hooks, APX dilator and Malyugin ring) to determine which design and deployment method are most likely to cause iris damage.

**Methods:**

We used a biofidelic 3-part iris FE model that consisted of the stroma, sphincter and dilator muscles. Our FE model simulated expansion of the pupil from 3 mm to a maximum of 6 mm using the aforementioned pupil expanders, with uniform circular expansion used for baseline comparison. FE-derived stresses, resultant forces and area of final pupil opening were compared across devices for analysis.

**Results:**

Our FE models demonstrated that the APX dilator generated the highest stresses on the sphincter muscles, (max: 6.446 MPa; average: 5.112 MPa), followed by the iris hooks (max: 5.680 MPa; average: 5.219 MPa), and the Malyugin ring (max: 2.144 MPa; average: 1.575 MPa). Uniform expansion generated the lowest stresses (max: 0.435MPa; average: 0.377 MPa). For pupil expansion, the APX dilator required the highest force (41.22 mN), followed by iris hooks (40.82 mN) and the Malyugin ring (18.56 mN).

**Conclusion:**

Our study predicted that current pupil expanders exert significantly higher amount of stresses and forces than required during pupil expansion. Our work may serve as a guide for the development and design of next-generation pupil expanders.

## Introduction

Cataract is clouding of the natural human crystalline lens. Its development is commonly associated with age and requires surgery to restore vision [1, 2]. These surgeries replace the lens with an artificial intraocular lens [3] and require an unobstructed sufficiently large pupil for surgical manoeuvres. Pharmacological eye drops such as phenylephrine and tropicamide are commonly used preoperatively and intraoperatively to relax the sphincter muscle and constrict the dilator muscle [4, 5]. As a result of aging, the pupil tends to constrict, which may be one of the factors leading to an increased incidence of complications. Other reasons for a small pupil even after attempted pharmacological dilation include: pseudoexfoliation [6, 7], an inflammatory membrane around the sphincter in uveitis [7], systemic drugs (e.g. tamsulosin [8]) and long term miotic drop usage (e.g. pilocarpine [7]). Even if initially adequate, the pupil may constrict later during phacoemulsification from iris trauma or prolonged operation time. Thus pupil expander devices are required in up to 3.2% [9] of cases to mechanically augment pharmacological dilation.

Several mechanical pupil expanders have been released onto the market [10, 11] including the Malyugin ring [12], the Assia Pupil Expander (APX), the Perfect Pupil [13, 14] and iris hooks [15–17]. The Malyugin ring engages the pupil at 8 separate locations, expanding it to an octagonal shape. The APX dilator uses a pair of scissor-like prongs to engage the pupil at 4 locations yielding a rectangular shaped pupil. Iris hooks, used as sets of 4 or 5 [15, 18], can expand the pupil to a quadrilateral or pentagonal shape.

Pupil expansion in this way elicits different magnitudes of mechanical stress on the iris that may also lead to surgical complications [14]. Iris hooks and the APX dilator require additional incisions to deploy and involve pulling the iris tissue with a small contact zone. This could potentially damage the pupil margin, resulting in sphincter tears and prolonged abnormal dilation [19]. This effect is more pronounced for the APX dilator where the two longer edges require more stretch than needed with iris hooks. The APX dilator could also slip from the injector resulting in sudden iris stretch. The Malyugin ring suffers from being caught in the pupil margin and furthermore, the ring could flip out of the iris plane to damage the cornea. Its deployment involves dramatic dragging of the iris to opposite ends to engage the pupil margin in cases of a very small pupil.

Currently, little research has been performed to fully understand the mechanical impact of these devices on the iris tissue. In this study, we aim to: (1) use computational modelling to estimate local iris stresses (i.e. internal forces) as a result of mechanical pupil expansion; and to (2) compare such stresses as generated from several commercially available expanders (iris hooks, APX dilator and Malyugin ring) to determine which design and deployment method are most likely to cause iris damage.

## Methods

In this study, we used the finite element (FE) method to predict the deformations and stresses exhibited by the iris tissue during mechanical pupil expansion. FE is a tool commonly used by engineers to model and optimize the design of complex mechanical structures by subdividing it into smaller, manageable elements and solving equilibrium equations to provide the distribution of the engagement of the structure in terms of stresses [20]. The use of FE has been extended to numerous areas of medicine [21]. including cardiology where heart rhythms and blood flow simulations can predict pathology changes and validate the design of heart valves [22]. FE simulations allow the flexibility to control *in vivo* parameters such as the intraocular pressure (IOP), boundary and loading conditions, iris stiffness, and iris geometry, which would otherwise be impossible experimentally. Specifically, we performed FE stress analysis of iris tissue during the deployment of iris hooks, the Malyugin ring, and the APX dilator; and we compared iris stress estimates across devices.

### 3D geometry of the iris tissue

FE simulations first require the definition of an iris geometry, to which boundary and loading conditions will be applied in subsequent steps. For consistency, the same iris geometry was used for all simulations.

**Fig 1:**
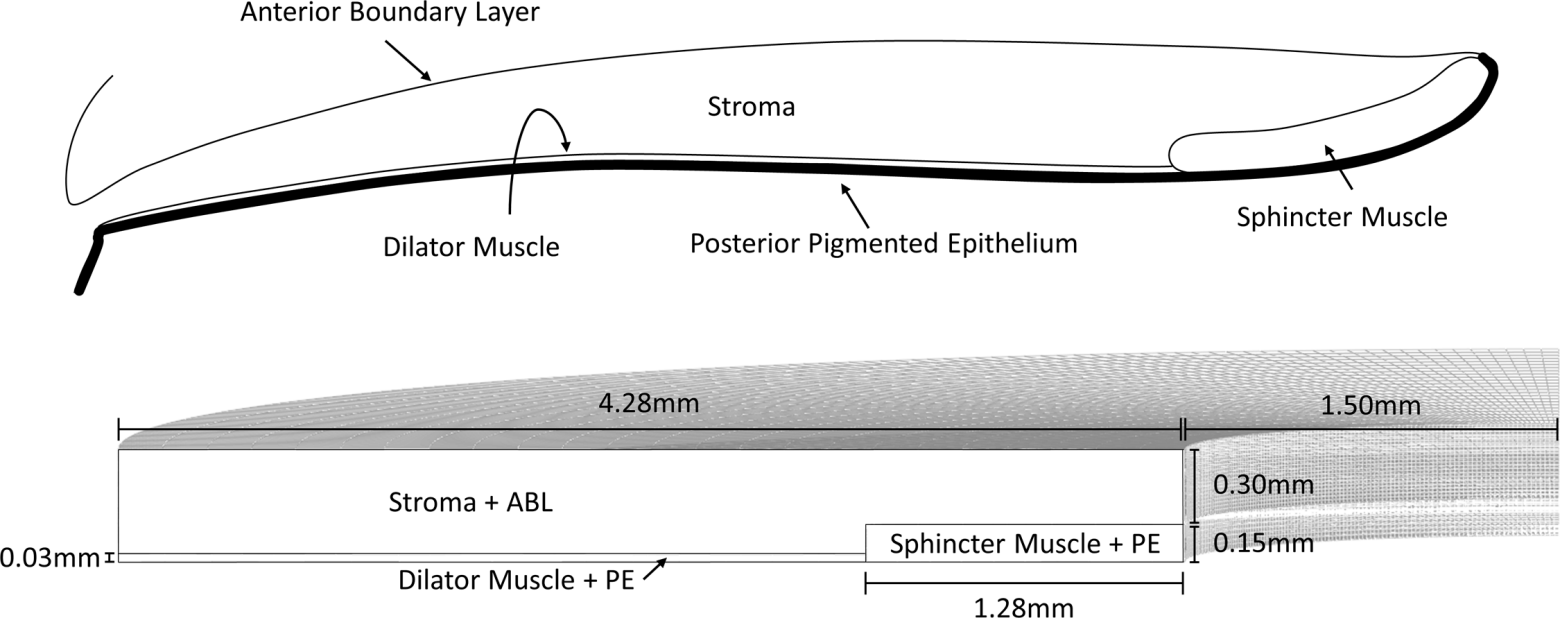
**A.** Anatomy of the iris tissue. The iris consists of the anterior boundary layer (ABL), the stroma, the sphincter and dilator muscles, and the posterior pigmented epithelium (PE). **B.** Geometry of the FE model used for simulations. The stroma and the ABL were combined into a single part, and the sphincter and dilator muscles were combined with the PE.

The iris is a soft tissue made up of several components. These distinct components include the anterior boundary layer, the stroma, sphincter and dilator muscles, and the posterior pigment epithelium. Within the stroma lies the vasculature and both myelinated and non-myelinated nerves that control accommodation of the iris muscles [23, 24] (Fig 1A).

The anterior boundary layer (ABL) is composed of the same cell types as the stroma, with the prior denser in cell count [23]. The stroma region is porous and mostly comprises of fibroblasts and melanosomes, making the bulk of the iris tissue [25]. These two layers were approximated as a single tissue in the FE model because the thin ABL is unlikely to provide any significant mechanical resistance during mechanical pupil expansion.

The sphincter muscle surrounds the pupil circumference and controls its constriction. Smooth muscle cells are innervated by nerve fibres from connective tissue within the stroma, similar to the dilator muscle at the posterior region of the iris [26]. The posterior pigmented epithelium is the final single cell layer of the iris. Its constituents are highly similar to those of the iris muscles [23]. Therefore, both layers were approximated as a single tissue in the FE model.

Although the iris is slightly elliptical, the differences in horizontal and vertical radii are not significantly different [27, 28] and can be approximated as circular. Therefore, the iris was modelled as a hollowed cylinder separated by 3 distinct tissues; the stroma, the sphincter muscle and the dilator muscle. Literature reports various ranges of anatomical measurements due to racial differences [29, 30], thus a consistent set of geometric values [24, 29, 31] within the range of reported measurements [24, 28–32] was selected for all simulations (Fig 1B).

The reconstructed iris was discretised into a hexahedron mesh with 50,176 elements. The mesh density was numerically validated through a convergence test involving models meshed with 2,944, 3,692, 7,168, 14,336, 50,176 and 93,184 elements, respectively. Convergence test results showed that the selected mesh density was within 3% of the most refined mesh and was deemed numerically acceptable. The FE iris geometry was designed and meshed within PreView (v1.17.2, Musculoskeletal Research Laboratories, University of Utah, UT, USA).

### Assigning iris biomechanical properties to the FE model

The iris stroma is a sponge-like tissue that is composed of 40% liquid and from which the aqueous humour can flow freely in and out during mydriasis and miosis [23, 25, 33, 34]. Therefore, the iris stroma was modelled as a biphasic material (i.e. 2 phases) and was composed of 1 solid matrix phase (60%; consisting of melanocytes, fibroblasts, blood capillaries and iris muscle nerves) and 1 interstitial fluid phase (40%) in order to take into account fluid exudation that is likely to occur during pupil expansion. Although stroma cells are not compressible, a biphasic material model would allow movement of aqueous humour, which would then allow for the compressibility of the iris stroma matrix. This phenomenon was observed *in vivo* in previous studies [35, 36]. Consequently, this could reduce the von Mises stress experienced by the iris stroma tissue. From the literature, the elastic modulus of the iris ranges from as low as 0.88 kPa (porcine data) [37] to as high as 6.2 kPa (extrapolated human modulus from bovine data at 9.6 kPa) [38]. Thus, an average elastic modulus (*E*) of 3 kPa and a Poisson's ratio (*v)* of 0.49 (to mimic incompressibility) were assigned to the solid matrix phase. Unfortunately, no research has been conducted to examine permeability of the iris. Therefore, permeability for the biphasic material was determined by variation of values to obtain optimal deformation shape of the iris. The permeability value used was 5 × 10^−6^ mm^4^/Ns (In contrast, the meniscus has permeability of 1.99 ± 0.79 × 10^−27^ mm^4^/Ns [39] due to slow exudation of fluid).

Both the dilator and sphincter muscles were modelled given the same material properties because of their similarity in tissue constituents. Muscle activity was not considered because pharmacological drugs (e.g. cyclopentolate and tropicamide) will induce cycloplegia. Therefore, the Ogden model was used to represent both muscles. It is a hyperelastic material model that describes non-linear stress-strain behaviours of complex tissues, able to capture the manner (shape) in which the iris tissue deforms with greater accuracy [40]. In the Ogden model, we used the biomechanical properties μ_1_, μ_2_, c_1_ and c_2_ which were equal to 54.3 (unitless), 48.1 (unitless), 0.1722 kPa and 0.1508 kPa, respectively [41]. These 4 parameters are material constants that describe the biomechanical behaviour of the iris tissue in the Ogden formula [42], described in the following hyperelastic strain energy function:

$$W\left(\lambda_{1},\lambda_{2},J\right)=\;\overset{N}{\underset{i=1}{\sum }}\frac{c_{i}}{\mu_{i}^{2}}\left({\tilde{\lambda }}_{1}^{\mu_{i}}+{\tilde{\lambda }}_{2}^{\mu_{i}}-3\right)+U\left(J\right)$$

where ${\tilde{\lambda }}_{i}$ are the deviatoric principal stretches and *c*_*i*_ and *μ*_*i*_ are the material parameters. The term *U(J)* is the volumetric component and *J* is the determinant of the deformation gradient.

### *In vivo* boundary conditions

**Fig 2:**
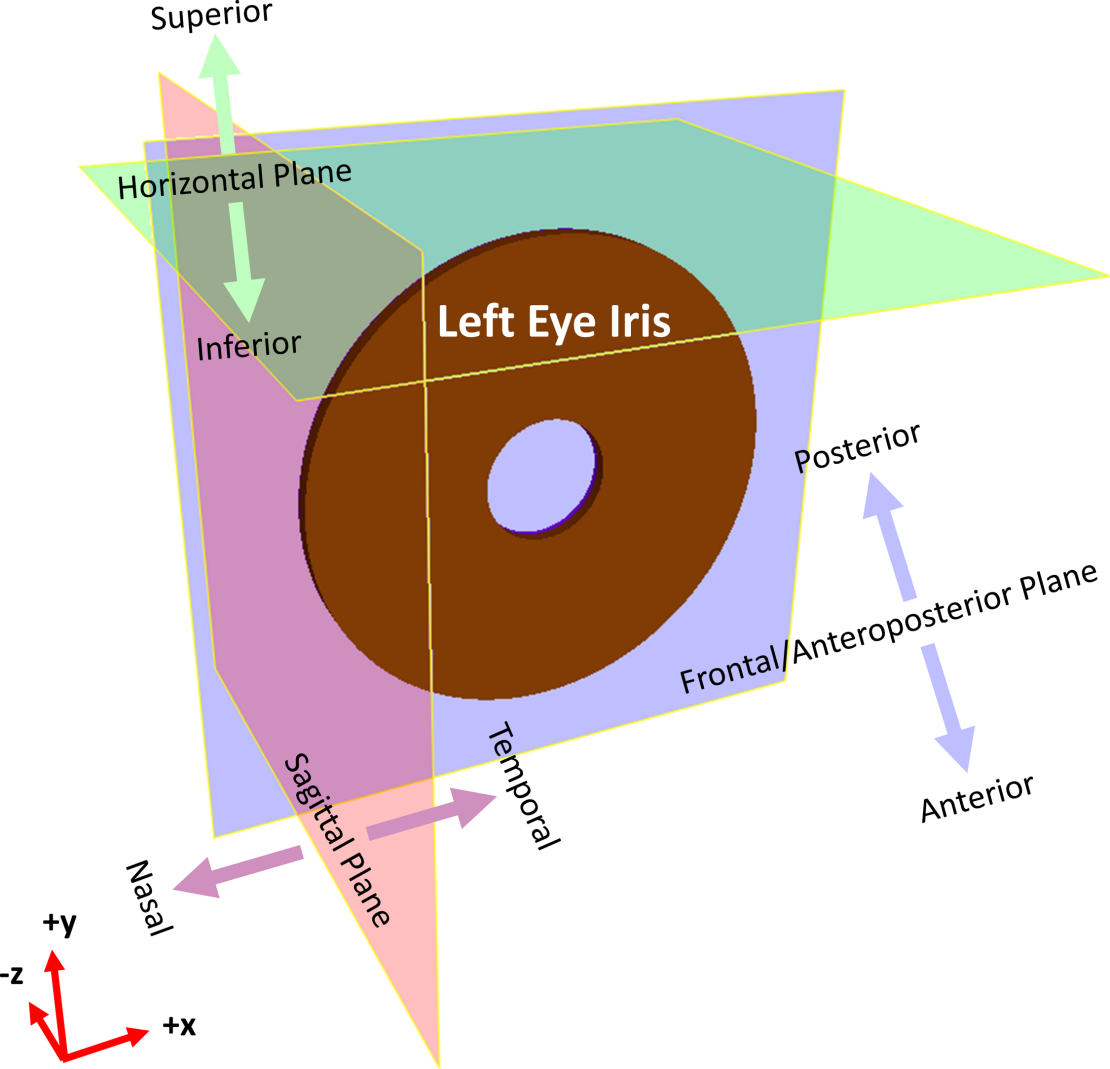
Anatomical orientation convention adopted in this study. The orientation adopted is in Cartesian coordinates in the *x*, *y* and *z* directions. The iris of a left eye is shown in this figure. The direction conventions of the arrows are perpendicular to their respective planes.

In order to mimic the iris attachment to the stable corneoscleral shell, the iris portion closest to the limbus was assumed to be fixed in place in the sagittal and horizontal planes during pupil expansion (i.e. displacements along *x* and *y* were set to zero). The iris was also allowed to thicken perpendicular to the frontal plane (i.e. free movement along *z*; refer to Fig 2 for the anatomical orientation terminology). During mechanical pupil expansion, the interstitial fluid of the iris was allowed to flow through the iris anterior surface to reach the anterior chamber of the eye. Thus, the pressure difference across this surface was set at *p* = 0 Pa.

### Mechanical pupil expansion–loading conditions in the FE models

Loading conditions for each pupil expander were applied separately depending on its design. Each model was subjected to mechanical loading from an initial pupil diameter of 3 mm to a maximum final diameter of 6 mm. It is important to emphasize that we only reported the stress state of the iris after each device has been fully deployed, and we did not report iris stress levels during the positioning of each device.

### Iris hooks

**Fig 3:**
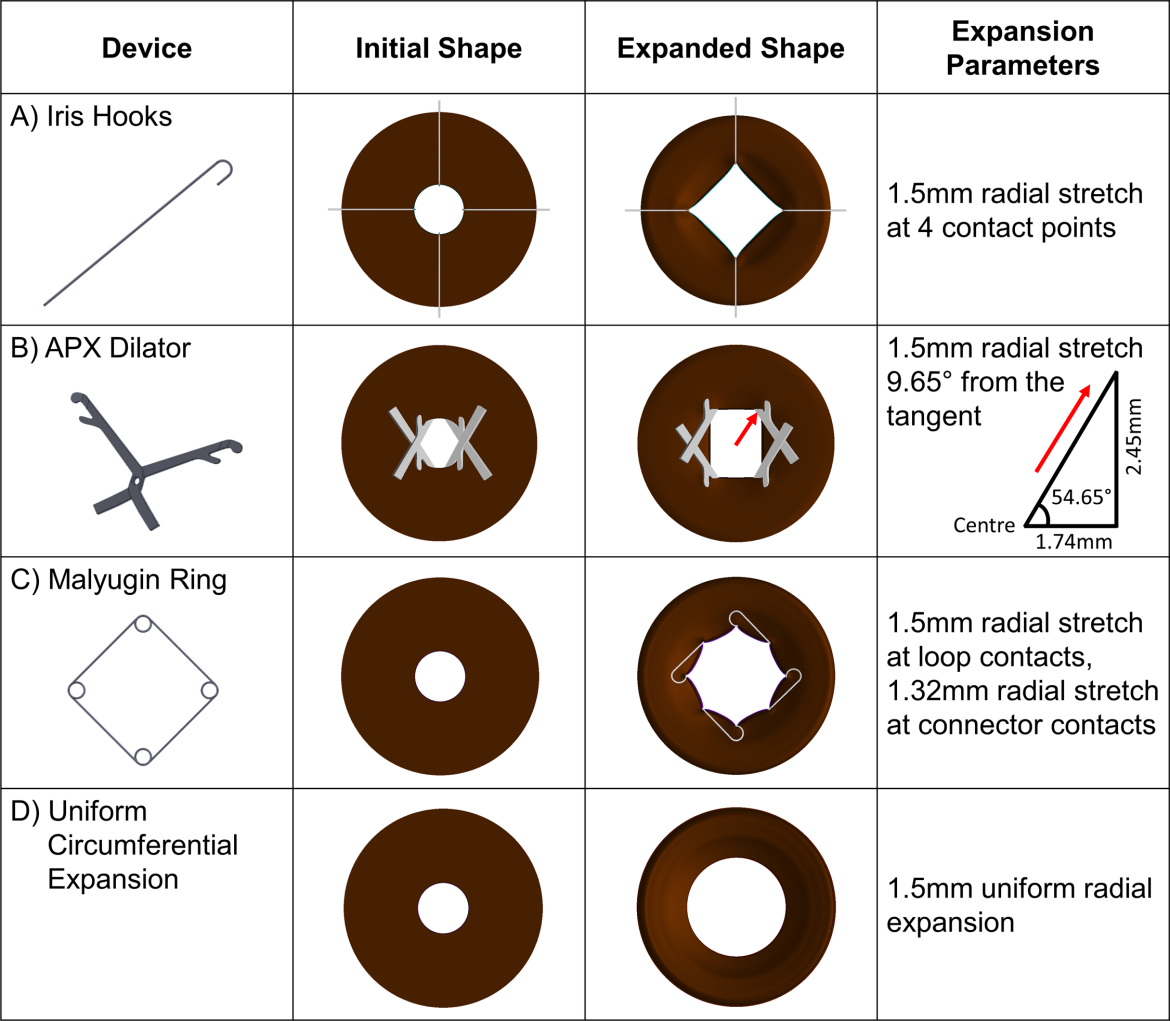
**The FE models and summary of the simulation parameters used in the analyses for A**. The iris hooks, **B.** The APX dilator, **C.** The Malyugin ring, and for **D.** Uniform circular expansion. All irides had an initial pupil diameter of 3 mm (initial shape). Following FE analysis, the deformed expanded pupil shapes had diameters of 6 mm (maximum observable diameter, expanded shape).

Iris hooks (S9-5014, FCI Ophthalmics, MA, USA) are cane-shaped devices with diameters of 0.08 mm. The 4 hooks are typically distributed evenly 90° from each other, giving a square- or diamond-shaped pupil opening. Insertion is through the cornea and retraction of the iris at an angle. This could require lifting the tissue upwards during expansion and making surgical access more difficult and exerting extra force in the *z* direction. Thus in the FE model, contact at 4 equidistant locations were made with a 3 mm pupil on the entire edge of the iris margin, in contact with both the stroma and the sphincter muscle. Displacement was performed 1.5 mm perpendicular to the iris margin to obtain a maximum square-shaped pupil of 6 mm at the orthogonal axes (Fig 3A).

### APX dilator

The APX dilator (APX-200, APX Ophthalmology Ltd., Haifa, Israel) has a scissor-like design with a strong flexible spring supplying the mechanical force to keep it apart. Two separate relatively large incisions are required to deploy the expander, inserting at an angle from the cornea similar to the iris hooks, in the closed position (in tension). Slow release of the specialised forceps engages the distal end curved pincer-like tips onto 2 points of the iris to stretch it, forming a non-physiological trapezoidal shape to increase space for surgical tools. This was simplified in the FE model to contact at 4 equidistant locations with a 3 mm pupil on the entire edge of the iris margin, in contact with both the stroma and the sphincter muscle. Displacement of 1.5 mm was applied at each location at an angle of 9.65° of 0.675 mm by 1.386 mm to give a final rectangular opening of 3.47 mm by 4.89 mm (Fig 3B).

### Malyugin ring

The Malyugin ring (MAL-0002, MicroSurgical Technology, WA, USA) has 4 corner helical loops to engage the iris. The straight connectors of these hooks also contact the iris at 4 additional diagonal locations to form an octagonal pupil. Deployment of the Malyugin ring involves placement of the helical hoops at the iris margin by engaging opposite portions of the iris, first the superior and inferior ends, then the nasal and temporal ends to counter the spring-like tension of the device. At the helical loops, the Malyugin ring expands the pupil to a larger diameter of either 6.25 mm or 7.00 mm. To offer consistency in the simulation parameters, these 4 locations were displaced 1.5 mm instead, from 3 mm to 6 mm perpendicular to the iris margin, in contact with both the stroma and the sphincter muscle. The 4 diagonal contact locations were displaced 1.32 mm perpendicular to the iris margin (Fig 3C).

### Uniform and circular pupil expansion

All models were benchmarked against a baseline model of uniform and circular pupil expansion. This expansion applies uniform force on the inner circumference radially. We believe such a scenario provides the optimal iris shape to limit tissue damage during pupil expansion due to even distribution of forces across the entire inner iris circumference. In the baseline model, the pupil was expanded uniformly from 3 to 6 mm (Fig 3D). This was done by applying a radial displacement of 1.5 mm to all points of the inner iris circumference edge.

All 4 FE models were solved using FEBio (v2.4.2 –Musculoskeletal Research Laboratories, University of Utah, UT, USA), a nonlinear FE solver designed for biomechanical studies [43]. FEBio is an open source software for nonlinear finite element analysis in biomechanics and biophysics. It offers various constitutive models and a wide range of boundary conditions to model biological interactions. This software [43] is well verified and has been used in more than 300 peer reviewed studies.

### Comparison of stress values

To evaluate and compare the various pupil expanders, we computed the stresses within the iris generated by each device from mechanical pupil expansion. Stress, measured in Pascals (Pa), is the force per unit area acting within the material, which balances the external applied forces. Strain is a normalized measure of deformation of an object compared to its original shape. Strain can be induced by many sources such as mechanical stress/force and temperature changes. In the case of stress/force induced strain, stress-strain relationship is defined by constitutive models of the material. The simplest relationship between stress and strain is linear elastic, in which strain is proportional to the stress level. High levels of stresses and strains can be found at the final shapes of the irides after the pupil expander has been fully deployed. The von Mises stress was the measurement of focus in these comparisons. Specifically, we reported von Mises stress (mean and peak) for the stroma and muscle sphincter regions induced by pupil expanders after complete deployment of each device. Von Mises stress is a scalar derived from principle stresses, which was used here as a convenient measurement to evaluate the stress levels of a tissue. However, it should be noted that it is still not clear which kind of stresses or strains has pathological relevance in iris tissues. Total area of the expanded pupil was also taken into consideration during the benchmark.

Calculation of von Mises stress values was conducted in PostView (v1.9.1, Musculoskeletal Research Laboratories, University of Utah, UT, USA). ImageJ [44] (v1.50i, National Institutes of Health, USA) was used to calculate and compare expanded pupil areas. The stresses were integrated over the corresponding surfaces of each element to obtain the force values. The forces of all evaluated elements were summed and reported as the reaction force.

### Comparison of reaction force values

Using our FE models, we also evaluated the forces required to expand the iris to the final shapes. Comparison with literature data was done to ensure that simulation results were valid (within the range of reported values). This was measured by addition of the resultant reaction forces of the iris nodes across the inner circumference that were in contact with the pupil expander.

## Results

Our FE models demonstrated that stresses created by pupil expanders were largely concentrated within the sphincter and dilator muscles of the iris (Table 1). The APX dilator generated the highest stresses at the contact points with the iris (max: 6.446 MPa; average: 5.112 MPa), followed by the iris hooks (max: 5.680 MPa; average: 5.219 MPa), and the Malyugin ring (max: 2.144 MPa; average: 1.575 MPa). On the other hand, uniform expansion generated the lowest stresses (max: 0.435 MPa; average: 0.377 MPa).

**Table 1:** Results of FE stroma and muscle stress values, and reaction forces experienced by the iris with the various pupil expanders.

	APX Dilator	Iris Hooks	Malyugin Ring	Uniform Expansion
Max Stroma Stress (MPa)	0.2722	0.1558	0.5163	0.0983
Average Stroma Stress (MPa)	0.2044	0.1338	0.2626	0.0899
Max Muscle Stress (MPa)	6.446	5.680	2.144	0.435
Average Muscle Stress (MPa)	5.112	5.219	1.575	0.377
Reaction Force (mN)	41.22	40.82	18.56	NA

A different trend was observed when comparing the stresses created by the pupil expanders within the stromal region. The Malyugin ring generated the highest stresses at the contact points with the iris (max: 0.5163 MPa; average: 0.2626 MPa), followed by the APX dilator (max: 0.2722 MPa; average: 0.2044 MPa), and the iris hooks (max: 0.1558 MPa; average: 0.1338 MPa). On the other hand, uniform expansion generated the lowest stresses (max: 0.0983 MPa; average: 0.0899 MPa).

**Fig 4:**
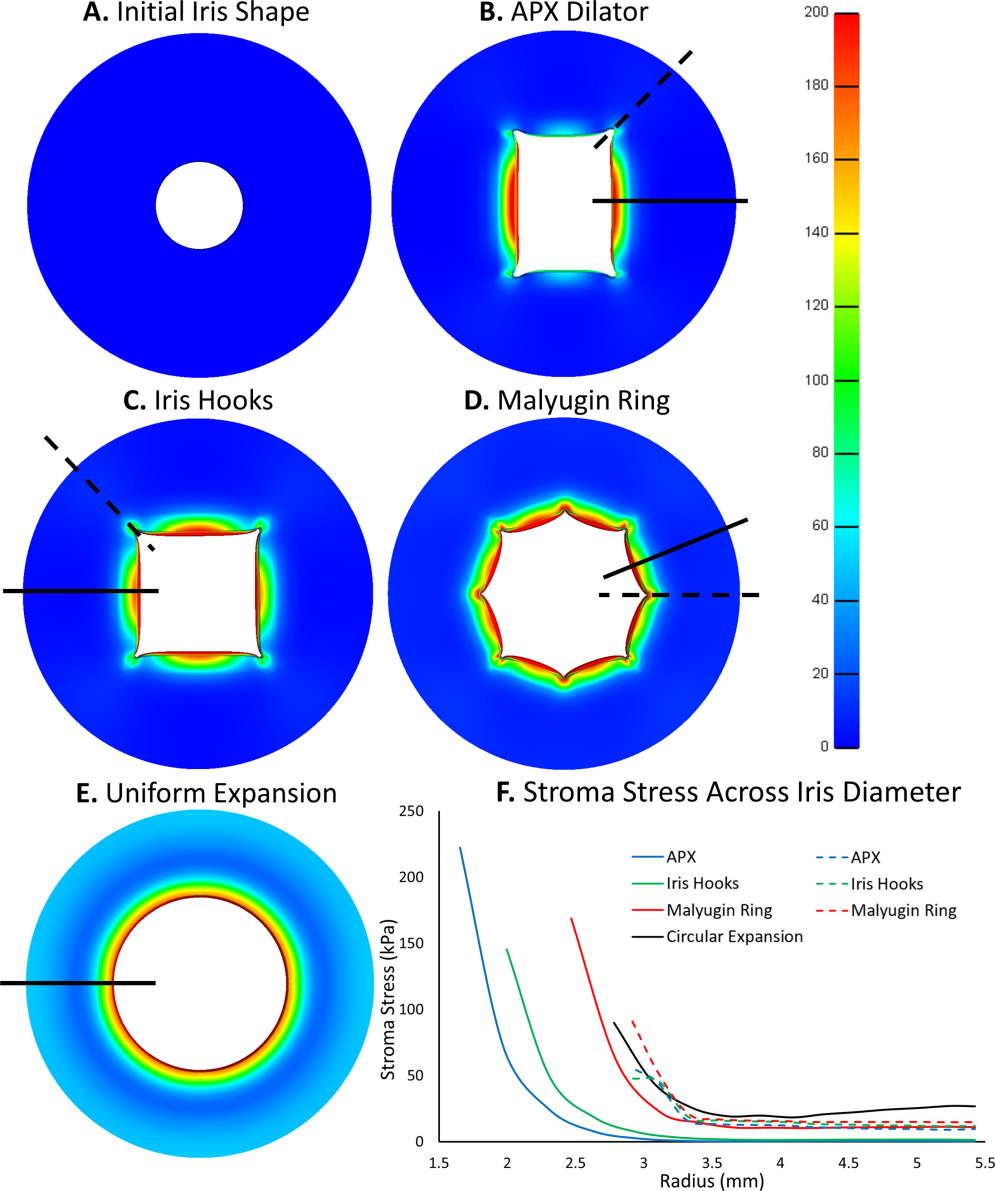
**A.** Initial iris shape with a pupil diameter of 3 mm before pupil expansion. Stress distribution of the iris tissue with the use of **B.** APX dilator, **C.** Iris hooks, **D.** Malyugin ring and **E.** Uniform circular expansion. The FE models were all deformed to a 6 mm pupil and stress magnitudes adjusted to the same scale. **F.** Graph of stromal stress along the radial direction starting at the point of greatest stress concentration (marked using solid black lines in **B-E**) and along the radial direction where a corner is present (marked using dotted black lines in **B-E**). Note that the starting point of each curve is determined by the size of the pupil and the ends at the iris limbus.

Stress distributions varied depending on the device. The APX dilator generated higher stress concentrations for larger areas (marked by larger red coloured areas in Fig 4B–4E) than the iris hooks and Malyugin ring at the inner iris circumference. Uniform expansion of the iris allowed it to distribute stresses (i.e. smaller stress gradient along the radial direction) more effectively at the iris margin (Fig 4F).

To open the pupil, the APX dilator required the highest force (41.22 mN), followed by iris hooks (40.82 mN) and the Malyugin ring (18.56 mN) (Table 1).

The final pupil opening was largest for uniform circular expansion (25.46 mm^2^), followed by the Malyugin ring (19.12 mm^2^), iris hooks (14.52 mm^2^) and the APX dilator (14.32 mm^2^).

## Discussion

In this study, we used computational modelling to estimate and compare local iris stresses (i.e. internal forces) as a result of mechanical pupil expansion and as generated from several commercially available expanders (iris hooks, APX dilator and Malyugin ring). We found that commercial pupil expanders generated high stresses within the iris stroma and dilator/sphincter muscles (due to unphysiological pupil shapes); and that such stresses were considerably higher than those exhibited during uniform circular expansion of the iris. All our stress analyses were performed using an improved and more biofidelic 3D model of the iris.

### Method of pupil expansion determines pupil opening size and iris stresses

Uniform circular expansion was used as a baseline, and the APX dilator performed the poorest in comparison. Specifically, despite having the smallest pupil opening (43.8% smaller), the APX dilator generated the largest stress on the muscle tissue (1255% greater) and required the most force to expand the pupil. Iris hooks fared slightly better than the APX dilator and the Malyugin ring performed the best amongst the 3 (refer to Table 2 for full comparison). Despite the Malyugin ring exerting the least stress among the 3 devices, uniform expansion offers 24.9% more surgical space yet causing 79.7% less stress. By distribution of force over the entire surface area of the sphincter muscle, circular expansion provides the physiological shape for pupil enlargement to reduce stress on the tissue. The physiological shaped opening could also prevent excessive post-operative deformation and reduced inflammation from the less damaged tissues.

**Table 2:** Comparison of all results with uniform circular expansion as the baseline. The sign (+) refers to “greater than” and the sign (-) refers to “smaller than”.

	Relative to Uniform Expansion		
	APX Dilator	Iris Hooks	Malyugin Ring
Max Stroma Stress	+ 177%	+ 59%	+ 425%
Average Stroma Stress	+ 127%	+ 49%	+ 192%
Max Muscle Stress	+ 1384%	+ 1207%	+ 393%
Average Muscle Stress	+ 1255%	+ 1283%	+ 317%
Area of Pupil	- 43.8%	- 43.0%	- 24.9%

### Uniform circular expansion generates the smallest stress gradients

While maximum stress values are typically correlated with tissue failure, large stress gradients have also been hypothesized to induce tissue and cell damage in soft tissues [45]. During mechanical pupil expansion, our FE models revealed that stress was typically concentrated near the margin of the iris, and decreased considerably moving toward the iris periphery (Fig 4B–4E). From our models, we extracted data to investigate how stress was distributed in the stroma along the radial direction (along the black lines shown in Fig 4B–4E). Steepness of the graph in Fig 4F represents the stress gradient across the tissue along the iris radius; starting points of the curves were determined by the size of the pupil opening at the inner circumference and at the point of maximum stress for all devices. The APX dilator and iris hooks had high stress gradients at the iris inner radius, but negligible stresses beyond 3.5 mm. The Malyugin ring fared slightly better, with an octagonal design than could distribute some stress within the peripheral stroma. Uniform circular expansion outperformed the rest, with the gentlest stress gradient that distributed much of the internal stresses towards the limbus. Even with the largest pupil opening, uniform expansion exhibited the lowest stress gradient through efficient stress distribution.

### FE analysis predicts greatest stresses within the sphincter muscle

Mechanical expansion of the iris generated stresses concentrated at the locations where the pupil expanders were in contact with the iris inner circumference. Our simulation results showed that stresses were largest (between 0.43 and 6.45 MPa) within the sphincter muscle for all pupil expansion scenarios. In contrast, stromal stresses were 1 order of magnitude lower (between 0.098 and 0.52 MPa). When comparing maximum stresses for each scenario, the magnitude of difference for stresses within the sphincter muscle (0.435 MPa to 6.446 MPa; 14.83 times) was larger than for stresses in the stromal region (0.098 MPa to 0.516 MPa; 5.25 times). By allowing fluid to escape during compression, it may be that the stromal tissue could become more compact without a large increase in internal forces. Several studies have shown that the iris acts like a sponge and during physiological dilatation may behave differently whether there is coexistent angle closure glaucoma or open angle glaucoma. The formation of small sphincter tears may alter the model too.

Even though FE analysis predicted that stresses exhibited by the sphincter muscles were greater than those by the stroma, iris damage may not occur at the sphincter first. The sphincter is made up of muscle fibres, which are tougher and can withstand larger stresses. Incidentally, the anisotropic nature of the circular fibres reduces its moduli against angular forces [46]. Depending on the method of pupil expansion, sphincter damage could occur at different stress values. However, due to large differences in generated stresses between the stroma and sphincter, failure at the sphincter could also result in failure at the stroma. Further experimental and histology studies are required to better understand how pupil expanders could damage muscle and stromal tissues in the iris.

### Softer pupil expanders with a circular expansion design should be prioritized

Surgeons have experienced pupil expanders that result in complications such as iris tearing, bleeding and intraoperative floppy iris syndrome (IFIS) [47–49], which could be attributed to the inefficient designs of existing pupil expanders. Uniform circular expansion of the iris allows for dilation of the pupil and stretch of the tissue in a physiological manner. It could reduce the chances of surgical complications to the iris through the use of softer materials. Existing materials for pupil expanders include polypropylene (iris hooks and Malyugin ring), stainless steel (APX-100) and rigid plastic (APX-200). These hard materials were required for exerting sufficient forces (33.7–56.5 mN) during pupil expansion. By changing the design to offer uniform expansion, these forces may be reduced significantly. Less force required allows for the possibility of adopting softer and more flexible materials (such as silicone) that may reduce tissue damage since they are more similar in mechanical properties to the surrounding tissues. Similarly, alternative designs which may engage larger segments of iris tissue may be beneficial.

### Our atress predictions were derived from a more biofidelic iris model

For this study, we used an improved iris model that may allow for a better representation of the characteristics of the iris behaviour. Instead of a using a singular structure iris model, we separated the iris into 3 distinct parts and attempted to represent each part more accurately based on existing literature.

Extensive research has been conducted on animal models of the iris. Heys and Barocas reported bovine elastic modulus of 9.6 ± 2.0 kPa for the iris dilator muscles [50]. Whitcomb et al. conducted radial and azimuthal extension of porcine irides and obtained the iris moduli of 4.0 ± 0.9 kPa and 2.97 ± 1.3 kPa, respectively [51]. Whitcomb et al. also performed nano-indentation on the anterior and posterior regions of porcine irides and derived instantaneous moduli of 4.0 ± 0.5 kPa and 6.0 ± 0.6 kPa respectively [52]. Lei et al. conducted experiments to obtain porcine elastic mechanical parameters of 5.3 N/m and 24.7 N/m in the radial and azimuthal directions respectively [38]. Beyond mechanical testing, non-linear hyperelastic models were also used in attempts to capture the viscoelastic behaviour or the iris. Jouzdani used the neo-Hookean solid model to simulate the iris, retrieving data of 1.1–4.0 kPa for the incompressible model and 0.88–1.43 kPa for the compressible model [37]. A more complex hyperelastic second order Ogden model was also used by Zhang et al. on rabbit irides to obtain material parameters of μ_1_, μ_2_, α_1_ and α_2_ of 86.1 kPa, 75.4 kPa, 54.3 and 48.1 respectively [41]. The correlation coefficient of curve fitting soft tissue experimental data conducted by Martins et al. showed that the Ogden model (correlation coefficient = 0.998) was more accurate than the standard neo-Hookean model (correlation coefficient = 0.957) [40]. Therefore, we adopted the Ogden model in our FE analysis to capture the deformation pattern of the iris during pupil expansion.

Several experiments were also conducted on post-mortem human eyes. Heys and Barocas extrapolated bovine data and estimated the human iris modulus to be 6.2 kPa, based on anatomical measurement differences between human and bovine irides [50, 53]. Tabandeh et al. conducted pharmacological treatment on donor cadaver eyes with pilocarpine and phenylephrine to induce sphincter and dilator muscle constriction [54]. The reported mean forces were 27.5 ± 5.7 mN and 23.3 ± 4.0 mN respectively, and 54.2 ± 6.6 mN when both drugs were combined. We were able to compare our FE analysis with these experimental data to verify that our simulation predictions were reasonable.

While many *ex vivo* experiments attempted to obtain the biomechanical properties of the iris, simple incompressible neo-Hookean assumptions [32] were insufficient to reflect the complexities of the iris under extreme deformations. The amount of strain exhibited by the iris during pupil expansion is over 100% from a 3 to 6 mm pupil. At the same time, the iris loses most of its volume [35, 36]. The way the iris easily deforms to a square pupil with the iris hooks is also not typically observed in other soft tissues. Therefore, to accurately perform FE analysis, we designed an iris model that incorporated experimental knowledge from the literature and that allowed: **1)** large volume changes during pupil opening; and **2)** accurate material models to capture iris deformation behaviours. The iris model was derived from anatomical measurements with 3 distinct segments: stroma, sphincter muscle and dilator muscle. The stroma was given biphasic properties with its porosity determined by anatomical microscopy research [23, 33]. The sphincter and dilator muscles were given biomechanical properties in an FE study from Zhang et al. [41] that tracked the movement of iris tissue during pupil dilation. Overall, our proposed approach was able to provide a ‘realistic’ deformation profile for the iris under extremely large deformations as experienced during pupil expansion.

### Limitations

First, our iris FE model was symmetrical and had a flat surface at the inner circumference. This created a large area of contact between the iris and the chosen pupil expanders. Furthermore, the human iris is not perfectly circular and symmetrical across any diameters [27], but these variations in dimensions may not be significant enough to result in major changes in stress. Further studies using patient-specific iris morphologies (as could be obtained with optical coherence tomography [55]) may be conducted to improve our stress predictions.

Second, the lens was not taken into account in our FE models. The iris is bowed forwards, i.e. slightly curved towards the anterior portion of the eye at the inner radius, and is thought to sit and slide over the lens during contraction. This nonplanar contour was not captured in our FE simulations, but shape differences should not be significant because the application of force was external and mechanical. The iris direction of motion was mostly based on the surgeon’s use and relative stresses should not differ greatly.

Third, FE analysis does not necessarily reflect the maximum stresses exhibited by the iris during mechanical pupil expansion. What we measured and compared were the final positions of the pupil expanders after deployment. The method of deployment varies with surgical manoeuvres which are difficult to predict. Iris hooks are usually deployed partially, then tightened by retracting the hooks further. However, accidental overtightening and then correction were not taken into account in the FE models. Some surgeons use 5 hooks instead of 4 for cataract surgery, and the exact positioning of these hooks are not perfectly equidistant, which may cause greater stress. Furthermore, the placement of iris hooks and the APX dilator may retract the iris upwards and not perfectly horizontally as these devices are attached to the limbus. The APX dilator is supposed to be deployed in a rectangular opening, with the longer parallel edge situated at the primary incision to allow greater surgical tools’ space. Instances of the deployment device slipping could also cause a sudden stretch of the iris resulting in iris tearing. The Malyugin ring’s deployment method may stress the iris significantly; it involves dragging the device to opposite margins in order to engage the loops. Logically, this effect could be more pronounced in subjects with smaller baseline pupil diameters. It would be additionally beneficial for surgeons to understand complications when using pupil expanders by exploring some of these surgical movements and how they affect iris stresses in future studies.

Fourth, there is a slight difference in parameters used for the Malyugin ring. In order to ensure that the different devices are compared fairly, the widest pupil diameter was standardized at 6 mm, whereas the Malyugin ring offers a 6.25 mm diameter variant. Thus, the stresses observed in the device would be slightly higher in reality, although the differences between devices should not differ significantly.

Fifth, there are human variations in iris biomechanical properties that were not accounted for. Race, age, pathology-related complications [56], and stromal permeability [57] are factors that could determine how much stress an individual’s iris can tolerate before injury. Our FE study was only able to quantify iris stresses and forces for a ‘normal’ human iris; Anatomical and physiological variations in human irides could yield larger or smaller percentage differences in stress across the mechanical expansion scenarios.

Sixth, we proposed an iris model that combined knowledge and biomechanical properties from various literature. Although our FE models produced forces that were in agreement with the literature (18.6–41.2 mN in our FE, up to 54.2 ± 6.6 mN for active muscle contraction) [54], there could still be errors from combining literature data that were obtained under different experimental conditions. For instance, the Ogden model [41] was derived from rabbit data, which was similar but not identical in representing the human iris [47, 58, 59]. The absence of anisotropy in the Ogden model also reflected a limitation in providing accurate biomechanical properties of the sphincter and dilator muscles. We were not able to incorporate iris muscle fibres’ orientation and concentration due to a lack of data in the literature. We hope to continue this work to incorporate anisotropy of muscle fibres in future models.

Finally, we proposed that softer pupil expanders with circular expansion design should be prioritized. However, from a clinical perspective, this could result in devices that introduce new challenges. For example, the Visitec i-Ring pupil expander (Item #587001, Beaver-Visitec International, MA, USA) requires step-by-step engagement of the iris margin at the distal end, proximal end and two lateral sides similar to the Malyugin ring, introducing excessive dragging that could be traumatic to the iris. The Morcher pupil dilator (MR-5S, FCI Ophthalmics, MA, USA) and Graether pupil expander (Graether 2000 Pupil Expander, Katena Products, Inc., NJ, USA) also require similar manipulations in the anterior chamber to position the devices. As a result of full iris margin engagement, the designs of these devices tend to be bulkier and are harder to deploy and remove.

## Conclusion

Our study predicted that current pupil expanders exert significantly higher amount of stresses than required during pupil expansion. Optimisations can be made to prevent excessive deformation and tissue damage, reducing the inflammatory response of patients’ tissues. Therefore, our work may serve as a guide for the development and design of next-generation pupil expanders. Further research is needed to refine the FE model to improve our stress predictions. Continued verification of the proposed model could be useful in understanding pathologies associated with the iris.

## Supporting information

S1 Fig Calculations for the required simulation parameters for the APX dilator (left) and Malyugin ring (right). (TIF)

S2 Fig Examples of pupil expander devices used by surgeons, which vary by region and institution. A quadrilateral or pentagonal pupil can be achieved with iris hooks, requiring a stab incision for each hook. The Malyugin ring provides 8 contact points for an octagonal pupil (middle), and the APX dilator requires two lateral incision to create a quadrilateral (rectangular or trapezoidal) pupil (right).
(TIF)

S3 Fig **A.** Geometry of the iris tissue and **B.** geometry of the FE model. The tissue is symmetric across the sagittal and horizontal planes but not across the frontal plane (red line). This was simplified in the FE model to be axis symmetric.
(TIF)

S4 Fig Deployment of the four iris hooks denoted in alphabetical order A, B, C and D. The stresses analysed for **S5 Fig** is indicated by the red arrows.
(TIF)

S5 Fig Stresses of the sphincter muscle at the red arrow locations from S4 Fig from the corresponding alphabetical image. The four hooks were deployed from 0–0.25, 0.25–0.5, 0.5–0.75 and 0.75–1 on the x-axis respectively. (*Note that the x-axis denotes arbitrary time units in a static finite element analysis.).
(TIF)

S1 File Excel file containing data sets. (XLSX)
