# Supplementary figures and images for: Numerical stress analysis of the iris tissue induced by pupil expansion: Comparison of commercial devices
Source: PLoS One. 2018 Mar 14;13(3):e0194141. doi: 10.1371/journal.pone.0194141 (PMC5851615; doi:10.1371/journal.pone.0194141)

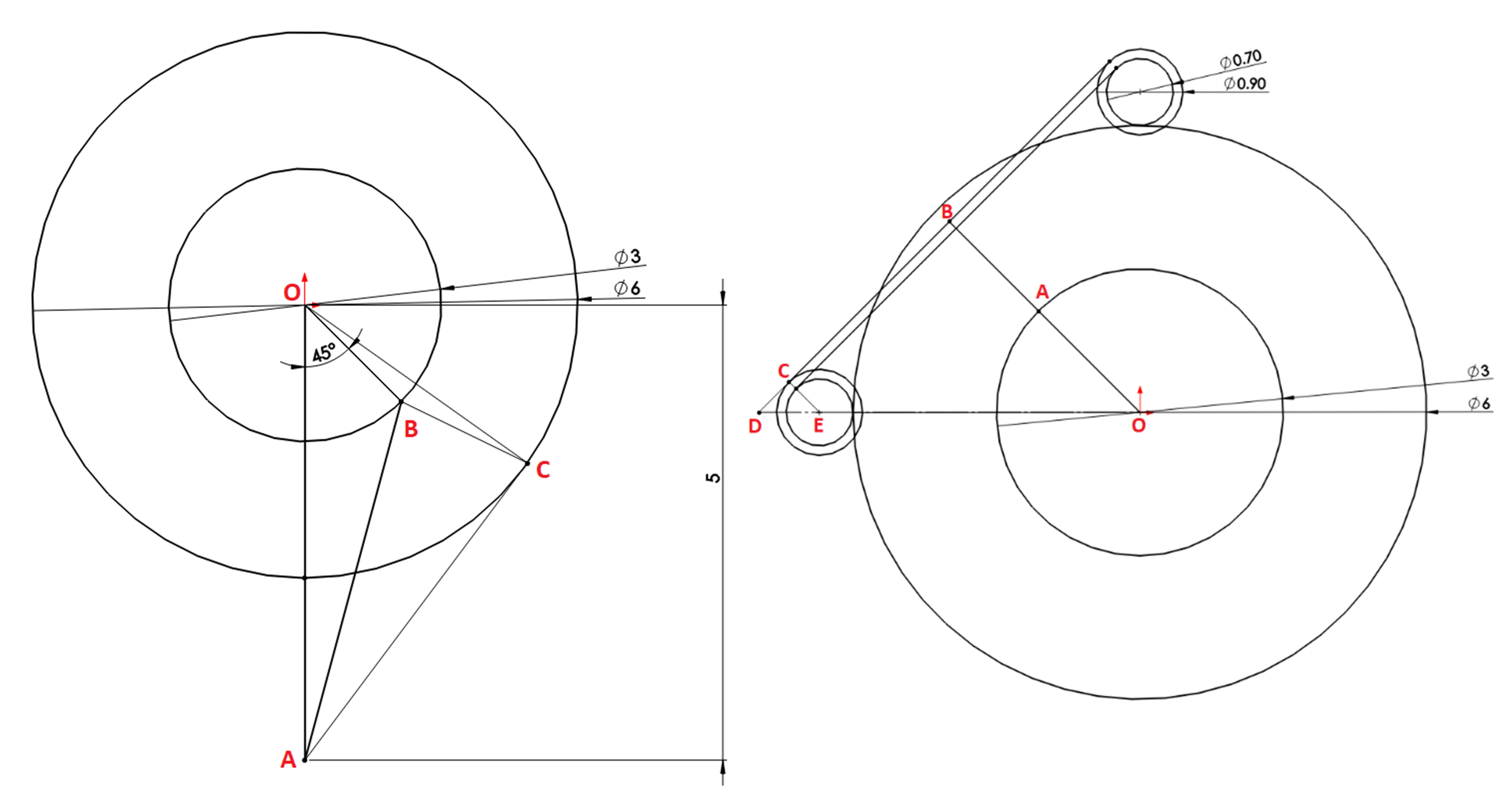

Supplement: S1 Fig — (TIF) [file pone.0194141.s001.tif]

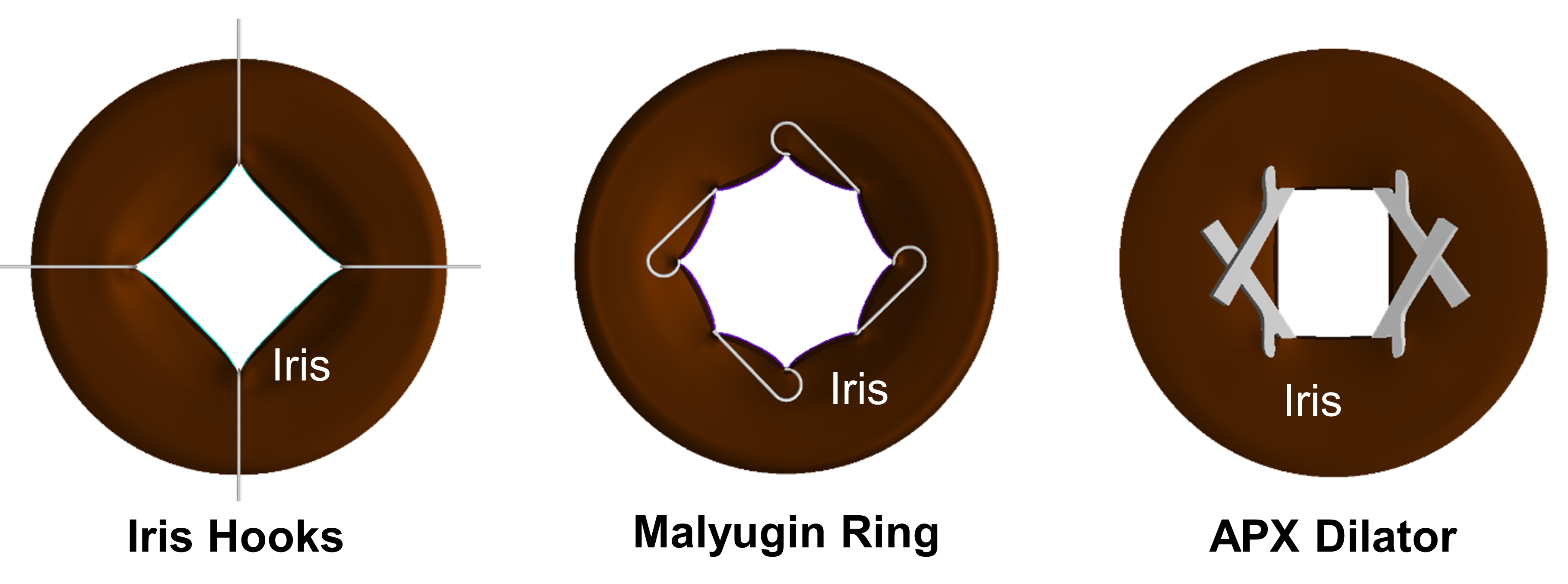

Supplement: S2 Fig — A quadrilateral or pentagonal pupil can be achieved with iris hooks, requiring a stab incision for each hook. The Malyugin ring provides 8 contact points for an octagonal pupil (middle), and the APX dilator requires two lateral incision to create a quadrilateral (rectangular or trapezoidal) pupil (right). (TIF) [file pone.0194141.s002.tif]

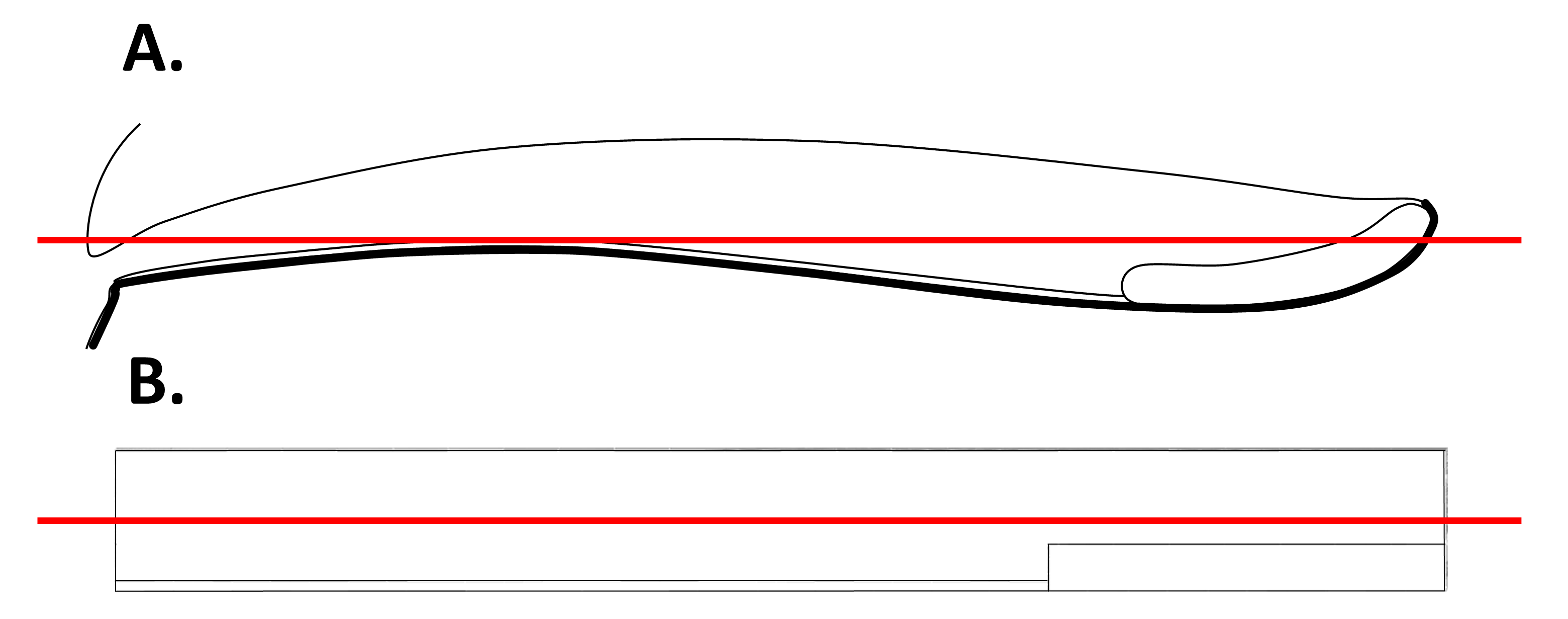

Supplement: S3 Fig — A. Geometry of the iris tissue and B. geometry of the FE model. The tissue is symmetric across the sagittal and horizontal planes but not across the frontal plane (red line). This was simplified in the FE model to be axis symmetric. (TIF) [file pone.0194141.s003.tif]

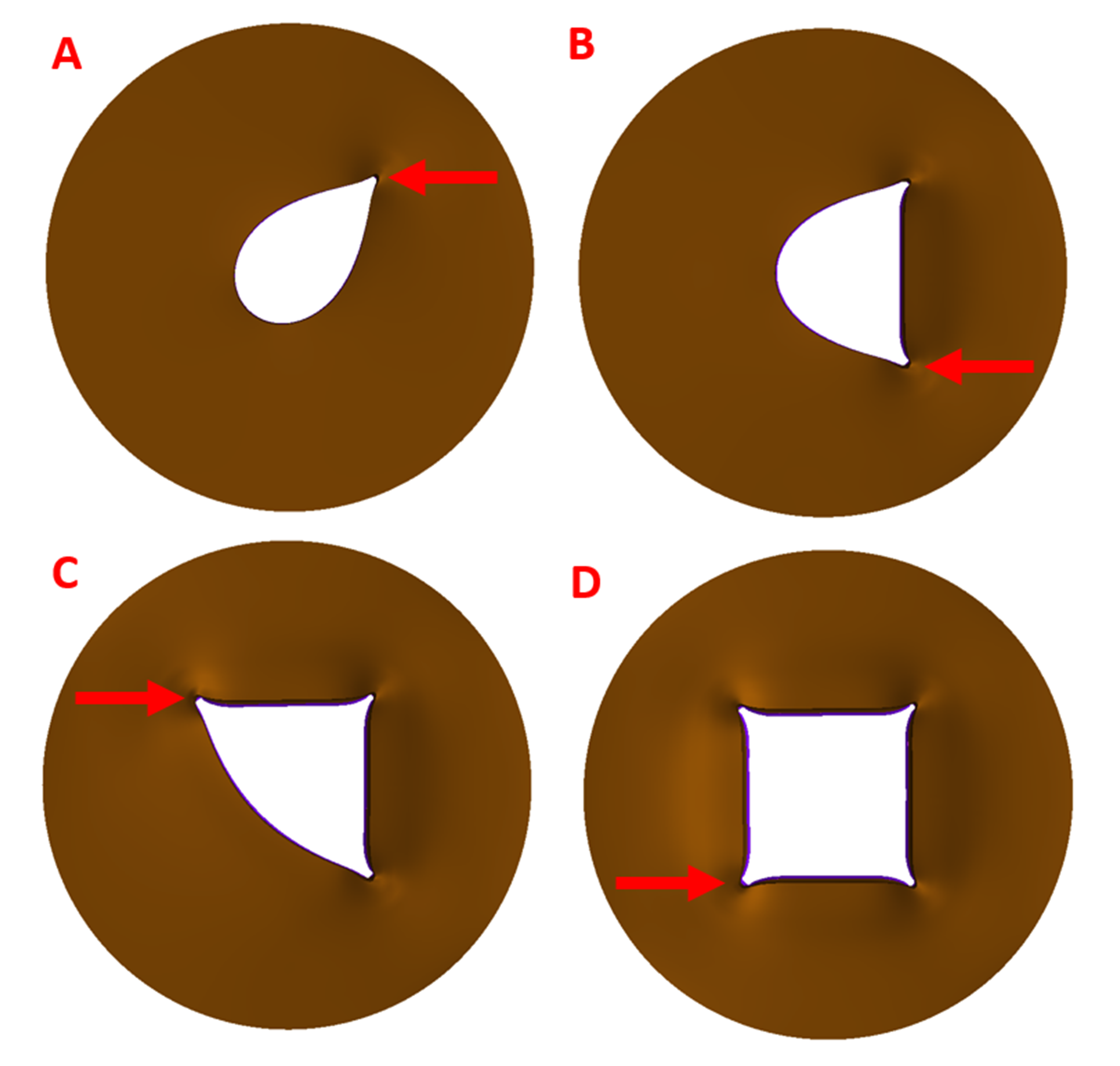

Supplement: S4 Fig — The stresses analysed for S5 Fig is indicated by the red arrows. (TIF) [file pone.0194141.s004.tif]

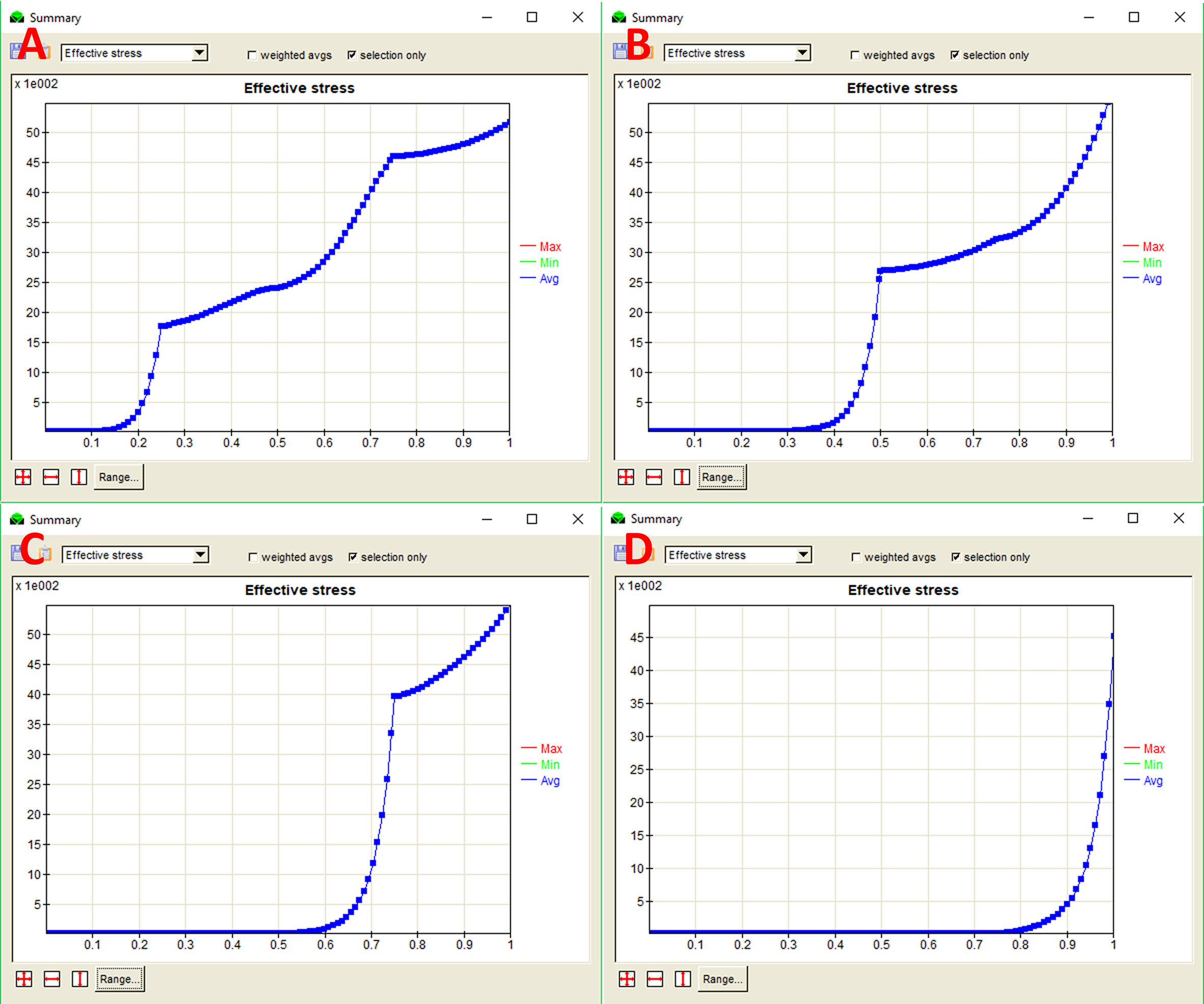

Supplement: S5 Fig — The four hooks were deployed from 0–0.25, 0.25–0.5, 0.5–0.75 and 0.75–1 on the x-axis respectively. (*Note that the x-axis denotes arbitrary time units in a static finite element analysis.). (TIF) [file pone.0194141.s005.tif]
